# Sertraline Pharmacokinetics in HIV-Infected and Uninfected Children, Adolescents, and Young Adults

**DOI:** 10.3389/fped.2019.00016

**Published:** 2019-02-06

**Authors:** Nathan John Hanan, Mary Elizabeth Paul, Yanling Huo, Suad Kapetanovic, Elizabeth Smith, George Siberry, Pim Brouwers, Bobbie Graham, Benjamin Johnston, Edmund V. Capparelli, Brookie M. Best

**Affiliations:** ^1^Department of Pediatrics-Rady Children's Hospital San Diego, University of California, San Diego, San Diego, CA, United States; ^2^Department of Pediatrics, Baylor College of Medicine, Houston, TX, United States; ^3^Center for Biostatistics in AIDS Research, Harvard T.H. Chan School of Public Health, Boston, MA, United States; ^4^Department of Psychiatry, University of Southern California, Los Angeles, CA, United States; ^5^Maternal, Adolescent, and Pediatric Research Branch, National Institute of Allergy and Infectious Diseases, Bethesda, MD, United States; ^6^Maternal and Pediatric Infectious Disease Branch, Eunice Kennedy Shriver National Institute of Child Health and Human Development, Bethesda, MD, United States; ^7^Division of AIDS Research, National Institute of Mental Health, National Institutes of Health, Bethesda, MD, United States; ^8^Frontier Science and Technology Research Foundation, Buffalo, NY, United States; ^9^Skaggs School of Pharmacy and Pharmaceutical Sciences, University of California, San Diego, La Jolla, CA, United States

**Keywords:** sertraline, pharmacokinetics, HIV, pediatrics, antiretrovirals

## Abstract

**Objective:** Due to potential disease and drug interactions, the appropriate sertraline starting dose and titration range may require adjustment in pediatric patients living with HIV. This is the first report of sertraline pharmacokinetics in HIV-infected youth.

**Methods:** IMPAACT P1080 was a multicenter pilot study describing psychiatric medication pharmacokinetics in HIV-infected and uninfected youth. Participants were stable on sertraline, >6 to <25 years old, and (1) HIV-uninfected (HIV(–)), (2) HIV-infected taking efavirenz (EFV), or (3) HIV-infected taking boosting ritonavir/protease inhibitor (PI/r). Sampling occurred at pre-dose, 2, 4, 6, 12, and 24-h post-dose. Analyses were performed for sertraline and N-desmethylsertraline, and CYP2D6 phenotyping was completed with dextromethorphan.

**Results:** Thirty-one participants (16 HIV(-), 12 PI/r, and 3 EFV) had median (range) weight, age, and dose of 69.5 (31.5–118.2) kg, 21.8 (9.1–24.7) years, and 75.0 (12.5–150.0) mg once daily. Sertraline exposure was highest for HIV(–) and lowest for EFV cohorts; median dose-normalized *AUC*_0−24_ was 1176 (HIV(–)), 791 (PI/r) and 473 (EFV) ng^*^hr/mL, and C_24_ was 32.7 (HIV(–)), 20.1 (PI/r), and 12.8 (EFV) ng/mL. The urinary dextromethorphan/dextrorphan (DXM/DXO) ratio was higher in HIV(–) vs. PI/r cohorts (*p* = 0.01). Four HIV(–) participants were CYP2D6 poor metabolizers (ln(DXM/DXO) of >-0.5).

**Conclusions:** HIV(–) cohort had the highest sertraline exposure. Sertraline exposure was ~40% lower in the PI/r cohort than in HIV(–); the need to alter sertraline dose ranges for PI/r participants is not clear. The impact of efavirenz on sertraline needs further investigation due to limited numbers of EFV participants.

## Introduction

The lifetime prevalence of major depression in patients with human immunodeficiency virus (HIV) is 22–45% (1), far exceeding the general population. Youth living with HIV are four times as likely to be treated with anti-depressant medication than uninfected youth (2). Two meta-analyses demonstrated a strong correlation between severity of depression and non-adherence to antiretroviral therapy (3, 4). Therefore, standard practice is to treat patients living with HIV and comorbid psychiatric conditions with psychotropic medications, despite a lack of adequate evidence regarding the risks. Psychiatric medications are often titrated to effectiveness. However, the typical starting dose and titration range may need to be adjusted in special populations (e.g., HIV and pediatrics) to produce exposures that have shown efficacy and tolerability in standard patient populations lacking comorbid conditions and interacting drugs.

Sertraline, a commonly used antidepressant, has an elimination half-life of about 27 h in adults, children, and adolescents (5). Sertraline undergoes extensive metabolism by cytochrome P450 (CYP) enzymes, CYP2B6, CYP2C9, CYP2C19, CYP2D6, and CYP3A4 (6, 7). N-demethylation forms N-desmethylsertraline, the major inactive metabolite. N-desmethylsertraline has an elimination half-life of 62–104 h (5). Genetic differences in metabolic pathways can lead to interpatient variability, with higher exposures in poor metabolizers and lower exposures in extensive metabolizers. A rough estimate of CYP2D6 intrinsic activity can be determined by administering a single dose of dextromethorphan and measuring the extent of metabolite (dextrorphan) formation compared to parent compound (a CYP2D6 phenotype assessment). Based on concentration ratio results, patients can be classified as either extensive or poor metabolizers. Numerous antiretrovirals are metabolized by and modulators of many of these same biotransformation pathways.

Ritonavir, a protease inhibitor and pharmacokinetic booster of other antiretrovirals, inhibits CYP3A4, the efflux transporter P-glycoprotein, and to a lesser extent, CYP2D6 (8, 9). However, at typical ritonavir boosting doses (100 mg), CYP2D6 inhibition is not clinically relevant (8). Ritonavir also induces CYP1A2, 2B6, 2C9, and 2C19, and the phase II uridine diphosphate-glucuronosyltransferase (UGT) enzymes (10–12).

Efavirenz, a common component of first-line antiretroviral therapy regimens, is a strong inducer of CYP3A4 and 2B6 (13). Efavirenz also induces CYP 2C19 in extensive and intermediate metabolizers, but not in CYP 2C19 poor metabolizers (14). Efavirenz decreases sertraline exposure (AUC_0−24_) by ~40% in adults. The induction of CYP 3A4 in particular, which has high concentrations in both hepatic and intestinal tissues, could contribute to decreased absorption through first-pass loss since sertraline is administered orally. Despite decreased sertraline exposure with efavirenz, no adjustment of starting dose is recommended (13).

In pediatric sertraline pharmacokinetic studies, sertraline exposure (AUC_0−24_ and C_max_) was ~22% lower in children and adolescents when plasma concentration was adjusted for weight (5, 9)/; other pharmacokinetic parameters were comparable to adults (15–19). In adolescents, sertraline kinetics are not linear; the half-life of sertraline shortens significantly (26.7–15.3 h) from single dose to steady-state (17). In addition, over a dose range of 50–200 mg, the half-life of sertraline at steady-state increases from 15.3–27.2 h (15, 17). The non-linearity of sertraline kinetics becomes increasingly important in youth with complex diseases and interacting medications. The objective of this study was to determine sertraline pharmacokinetics in youth without HIV, or with HIV and taking either an efavirenz-based or a ritonavir-boosted protease inhibitor-based regimen.

## Materials and Methods

International Maternal Pediatric Adolescent AIDS Clinical Trials Network (IMPAACT) P1080 was a multicenter, pilot study of psychiatric and antiretroviral medication concentrations in HIV-1-infected and uninfected youth. Target enrollment for the sertraline study arm was 45 participants >6 to <25 years of age. 45 participants had sufficient power to detect a 50% difference in apparent oral clearance (CL/F) among HIV-infected and uninfected cohorts, assuming a coefficient of variation of 30%. Participants were divided into three cohorts: (1) HIV-uninfected (HIV(-)), (2) HIV-infected taking concomitant efavirenz (EFV), and (3) HIV-infected taking boosting ritonavir with a protease inihibitor (PI/r): protease inhibitors could include atazanavir, darunavir, fosamprenavir, indinavir, lopinavir, saquinavir or tipranavir.

All participants gave signed informed consent or assent, and parents or guardians gave signed informed permission in accordance with the Declaration of Helsinki and local guidelines. The protocol was approved by human subjects' protection committees or institutional review boards at each participating site (see Acknowledgments for a listing of participating sites). Participants took sertraline for clinical care for at least 2 weeks prior to enrollment. Participants who were pregnant or taking interacting illicit drugs were excluded.

Study procedures were standardized across multiple study arms. The single pharmacokinetic visit included a medication history, adherence survey, and a CYP2D6 phenotype assessment (the other psychotropic study medications were primarily metabolized by CYP 2D6). Participants with HIV had to be taking their antiretroviral medications consistently for at least 4 weeks prior to sampling. The visit was scheduled so that a witnessed dose of sertraline and antiretroviral medications occurred on time, according to the participant's dosing regimen. Plasma samples were drawn at pre-dose and 2, 4, 6, 12, and 24-h post-dose.

A liquid chromatography-electrospray ionization-mass spectrometry method quantitated sertraline and N-desmethylsertraline. Isotope-labeled sertraline was used as an internal standard. Analytes and internal standard were extracted with 600 μL of acetonitrile from 200 μL of plasma, then eluted from a Gemini C18 column in under 4 min using acetonitrile/water/formic acid (85:15:0.1, v/v/v) as the mobile phase. Quantitation was performed with selective reaction monitoring of the transitions of m/z 306.2 → 159.1 for sertraline, 292.2 → 275 for N-desmethylsertraline, and 309.2 → 159.1 for internal standard. The method was linear over the concentration range of 1–320 ng/mL for both parent and metabolite.

The ratio of dextromethorphan (DXM)/dextrorphan (DXO) in urine was measured using a validated LC-MS/MS method following a single oral dose of dextromethorphan cough syrup (15 mg for participants ≥6 to <12 years old; 30 mg for participants ≥12 to <25 years old). Poor metabolizer phenotype was defined as urinary DXM/DXO molar ratios > 0.3 or log ratios > −0.5, while extensive metabolizer phenotype was defined as molar ratios ≤ 0.3 or log ratios ≤ −0.5.

Plasma concentrations of primary protease inhibitors, ritonavir and efavirenz were measured at the University of California San Diego Pediatric Pharmacology Laboratory by validated reverse phase high performance liquid chromatography or mass spectrometry methods. The interassay coefficients of variation were all <18%, and the mean recovery from the plasma ranged from 98 to 117%. The lower limits of quantitation were: 0.047 μg/mL for atazanvir, 0.090 μg/mL for darunavir, 0.091 μg/mL for lopinavir, 0.094 μg/mL for ritonavir, and 0.039 μg/mL for efavirenz.

Plasma concentration at pre-dose (C_0_), the maximum concentration (C_max_), the corresponding time (t_max_), and the concentration at 24 h post-dose (C_24_) were identified by direct inspection of concentration-time curves. The area under the concentration vs. time curve from time 0–24 h post dose (AUC_0−24_) was estimated using the trapezoidal rule. Apparent oral clearance (CL/F), where F is bioavailability, was calculated as dose divided by AUC_0−24_. The apparent volume of distribution (V/F) was determined as CL/F divided by k, where k is the terminal slope of the log plasma concentration-time curve. The half-life (t_1/2_) was calculated as 0.693/k. All concentration data were normalized to dose (100 mg) and to weight (70 kg). Non-compartmental parameters were estimated with Phoenix® WinNonlin® (Certara L.P. (Pharsight), St. Louis, MO). Pharmacokinetic parameters were compared between HIV(–) and PI/r cohorts with Wilcoxon rank-sum tests, two-sided with significance set to 0.05. Statistical comparisons were not made with the EFV group due to the low enrollment/accrual in this cohort (*n* = 3).

Sertraline population pharmacokinetics were evaluated using non-linear mixed-effects modeling (NONMEM, version 7.4). A one-compartment model at steady-state with first-order absorption and elimination best described the data (ADVAN2 TRANS2, FOCE with interaction). A combined (additive and proportional) residual error model was used. Covariates were screened individually on each pharmacokinetic parameter (CL/F, V/F, and k_a_). For all models, goodness of fit were assessed with diagnostic plots. All covariates that improved model fit at *p* < 0.05 were included in the multivariate screen. The multivariate screen removed one covariate at a time, until every combination of covariates that were significant in the univariate screen were tested; covariates were retained if, when removed from the model, the model significantly worsened at *p* < 0.01.

## Results

Thirty-one participants completed pharmacokinetic visits (*n* = 16 HIV(–); *n* = 3 EFV; *n* = 12 PI/r: 5 on atazanavir/ritonavir, 5 on darunavir/ritonavir, and 2 on lopinavir/ritonavir). The median weight and height of participants on the day of sampling were 69.5 kg and 167.2 cm, respectively (Table 1). The median age was 21.8 years (range 9–24.7). Participants' daily sertraline doses ranged from 12.5 to 150 mg. Median weight-normalized dose in HIV(–) (1.3 mg/kg) was higher than in both the PI/r and EFV groups (0.9 and 0.7 mg/kg; Table 1). A total of 181 plasma concentrations were measured. Two participants did not return for their 24-h time points, while three participants took their next dose of sertraline prior to the 24-h blood draw. Pharmacokinetics were estimated based on the pre-dose through 12 h post-dose concentrations for these participants.

**Table 1:** Participant demographics, Median (Interquartile Range)^a^.

	**HIV(-)**	**PI/r**	**EFV**
	(*n* = 16)	(*n* = 12)	(*n* = 3)
Weight (kg)	65 (58, 77)	73 (69, 77)	58 (45, 82)
Height (cm)	166 (163, 172)	169 (165, 175)	152 (145, 165)
Weight Normalized Daily Dose (mg/kg)	1.3 (0.9, 1.5)	0.9 (0.6, 1.4)	0.7 (0.6, 1.1)
Age (years)	22.8 (18.2, 23.3)	21.8 (20.9, 22.7)	19.3 (14.2, 19.5)
**SEX (%)**			
Female	10 (62.5)	8 (66.7)	2 (66.7)
Male	6 (37.5)	4 (33.3)	1 (33.3)
**RACE (%)**			
American Indian	1 (6.2)	0 (0.0)	0 (0.0)
Asian	1 (6.2)	0 (0.0)	0 (0.0)
Black	2 (12.5)	11 (91.7)	2 (66.7)
Unknown	0 (0.0)	1 (8.3)	0 (0.0)
White	12 (75.0)	0 (0.0)	1 (33.3)

a *For the EFV cohort, median (minimum, maximum) are reported*.

**Figure 1:**
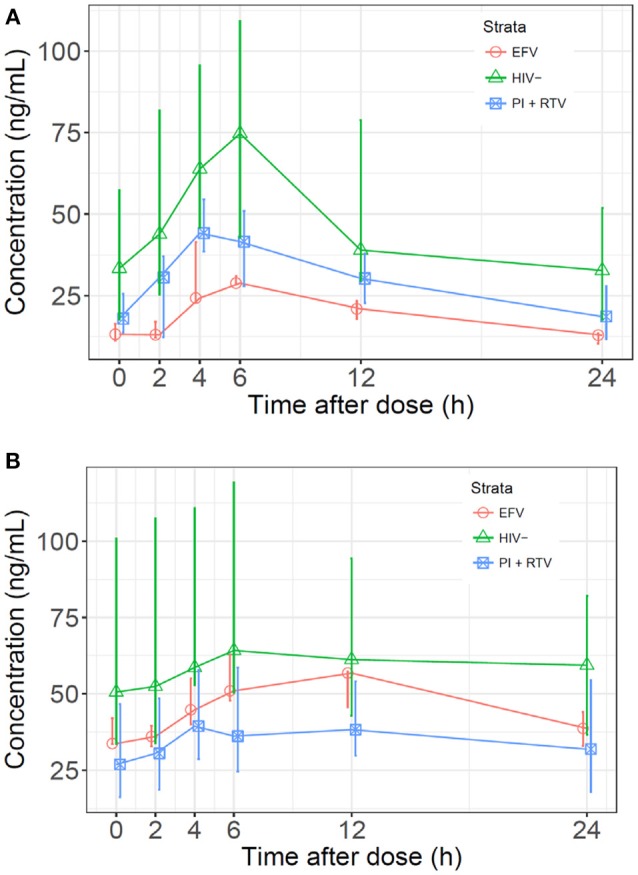
**(A)** *Median sertraline profiles by cohort*. Sertraline concentrations were normalized to a dose of 100 mg and a patient weight of 70 kg. **(B)** *Median N-desmethylsertraline profiles by cohort*. Concentrations were normalized to a sertraline dose of 100 mg and a patient weight of 70 kg.

Median normalized AUC_0−24_ values were 1176, 791, and 473 ng·h/mL for HIV(–), PI/r and EFV cohorts, respectively (Table 2 and Figure 1). Non-compartmental oral CL/F were not different in the HIV(–) and PI/r groups (1.4 vs. 1.6 L/h/kg, *p* = 0.59). However, CL/F was markedly higher in the EFV group (4.5 L/h/kg). Of C_0_, C_max_, and C_24_, only C_0_ was significantly higher in the HIV(–) compared to the PI/r cohorts (unadjusted and dose-normalized, *p* = 0.03).

**Table 2:** Sertraline and N-desmethylsertraline pharmacokinetic parameters, median (Interquartile Range)^a^.

**Parameter**	**HIV(-) *n* = 16**	**PI/r *n* = 12**	***p*-value^**b**^**	**EFV *n* = 3**
**SERTRALINE**				
*AUC_0−24_*(ng*hr/mL)	652 (506, 1407)	486 (415, 670)	0.08	145 (138, 286)
Norm-*AUC_0−24_*(ng*hr/mL)^c^	1176 (713, 1890)	791 (546, 961)	0.12	473 (374, 614)
*C_0_* (ng/mL)	20.1 (12.6, 39.7)	10.0 (7.5, 15.9)	0.03	6.0 (3.0, 7.0)
Norm-*C_0_*(ng/mL)^c^	33.3 (17.6, 57.3)	17.8 (9.9, 23.0)	0.03	13.1 (9.2, 19.6)
*C_*max*_* (ng/mL)	46.7 (36.5, 90.1)	34.3 (23.6, 41.7)	0.09	13.2 (8.8, 22.1)
Norm-*C_*max*_* (ng/mL)^c^	78.3 (50.9, 110.7)	46.9 (42.2, 68.7)	0.06	28.8 (28.4, 58.7)
*T_*max*_* (hr)	4 (4, 6)	4 (4, 6)	1.00	6 (4, 6)
*C_24_* (ng/mL)	17.5 (14.3, 40.1)	12.6 (8.6, 18.9)	0.07	4.2 (2.9, 5.9)
Norm-*C_24_* (ng/mL)^c^	32.7 (17.6, 51.9)	20.1 (11.8, 29.2)	0.17	12.8 (7.6, 13.8)
*CL/F* (L/hr/kg)	1.4 (0.8, 2.3)	1.6 (1.2, 2.3)	0.59	4.5 (1.6, 11.5)
*T_1/2_* (hr)	26.4 (14.1, 35.3)	18.1 (12.5, 23.1)	0.28	11.1 (10.2, 20.7)
*AUC* Ratio (DSRT/SRT)	1.4 (1.2, 1.7)	1.3 (0.7, 1.6)	0.13	2.2 (2.1, 2.6)
Ln(DXM/DXO)^d^	−2.3 (−3.0, −0.6)	−4.3 (−4.8, −3.8)	0.01	−2.35
**N-DESMETHYLSERTRALINE**				
*AUC_0−24_*(ng*hr/mL)	1063 (848, 1721)	670 (230, 1244)	0.09	376 (293, 624)
Norm-*AUC_0−24_*(ng*hr/mL)^c^	1533 (1053, 2133)	899 (646, 1186)	0.01	1223 (814, 1301)
*C_0_* (ng/mL)	41.7 (29.2, 65.8)	17.8 (4.8, 43.1)	0.05	15.5 (7.6, 25.5)
Norm-*C_0_* (ng/mL)^c^	50.6 (33.5, 100.9)	23.8 (11.7, 45.0)	0.01	33.7 (33.2, 50.3)
*C_*max*_* (ng/mL)	56.3 (45.6, 92.0)	34.4 (12.5, 57.6)	0.07	17.5(16.8, 34.1)
Norm-*C_*max*_* (ng/mL)^c^	76.6 (54.1, 138.7)	44.6 (33.1, 58.8)	0.01	56.8 (44.5, 74.7)
*C_24_* (ng/mL)	40.8 (30.4, 68.3)	20.5 (10.3, 45.1)	0.07	15.2(8.7, 20.8)
Norm-*C_24_* (ng/mL)^c^	59.4 (36.6, 82.0)	33.6 (17.8, 56.4)	0.03	38.7 (27.1, 49.3)

a *For the EFV cohort, median (minimum, maximum) are reported*.

*^b^ p-values calculated using the Wilcoxon rank-sum test for HIV(–) and PI/r comparisons*.

c *Normalized to a 100 mg once daily dose and a weight of 70 kg*.

d *Urine DXM/DXO ratio was measured in 12 HIV(–), 6 PI/r, & 1 EFV*.

Sertraline typical population pharmacokinetic values for CL/F, V/F, and k_a_ were estimated for the structural base model at 4.77 L/hr/kg^0.75^, 53.8 L/kg, and 0.45 hr^−1^ respectively (Table 3). The final population model (objective function decreased by 8.053 compared to the base model), which included the effect of age on clearance, resulted in estimated population values for CL/F, V/F, and k_a_ of 4.35 L/hr/kg^0.75^, 44.6 L/kg, and 0.42 hr^−1^.

**Table 3:** Base & final population pharmacokinetic parameters^a^.

**Parameters**	**Base model**	**Estimate (% SE)**	**Final model**	**Estimate (% SE)**
*CL/F* (L/hr/kg^0.75^)	θ_1_*ALWT^0.75^exp(η_1_)	4.77 (12)	θ_1_*ALWT^0.75^exp(η_1_)*((Age/21.8)exp(θ_4_))	4.35 (10.6)
*V/F* (L/kg)	θ_2_*WT	53.8 (20.1)	θ_2_*WT	44.6 (10.3)
*ka* (1/h)	θ_3_	0.45 (16.2)	k + θ_3_	0.42 (14.7)

a *ALWT, allometric weight; WT, weight; η_1_, inter-individual variability on clearance; θ_1_, typical population estimate for CL/F; θ_2_, typical population estimate for V/F, θ_3_, typical population estimate for ka. K, elimination rate constant*.

Median normalized N-desmethylsertraline AUC_0−24_ in the PI/r group was significantly lower than in the HIV(-) group (899 vs. 1,533 ng·h/mL, *p* = 0.01; Table 2 and Figure 1). Following normalization, median C_max_ in HIV(–) was statistically higher than PI/r (76.6 vs. 44.6 ng/mL, *p* = 0.01). Following normalization, C_0_ and C_24_ in HIV(–) were statistically greater than PI/r (50.6 vs. 23.8 ng/mL, *p* = 0.01; 59.4 vs. 33.6 ng/mL, *p* = 0.03).

Median (interquartile range) ritonavir AUC_0−12_ and C_max_ for PI/r participants were 4.9 (3.0, 9.4) μg·h/mL and 0.6 (0.4, 1.6) μg/mL (Table 4). Median (range) efavirenz AUC_0−24_ and C_max_ for the three EFV participants were 24.5 (24.4, 68.2) μg·h/mL and 1.3 (1.3, 3.4) μg/mL. No relationship was observed between ritonavir AUC_0−12_ and dose normalized sertraline AUC_0−24_ (*r*^2^ = 0.005) or ritonavir AUC_0−12_ and the AUC_0−24_ ratio of N-desmethylsertraline/sertraline (*r*^2^ = 0.09). No correlation was present between efavirenz AUC_0−24_ and dose-normalized sertraline AUC_0−24_ (*r*^2^ = 0.01). A correlation was present between efavirenz AUC_0−24_ and the AUC_0−24_ ratio of N-desmethylsertraline/sertraline (*r*^2^ = 0.97). However, with a small sample size (*n* = 3), these results should be interpreted with caution.

**Table 4:** Antiretroviral exposures, median (Interquartile range)^a^.

	***n***	*AUC_0−*tau*_* (μg*hr/mL)	***C_*max*_* (μg/mL)**	***C_*last*_* (μg/mL)**
**ANALYTE**				
Atazanavir	5	27.2 (21.8, 38.7)	3.8 (3.1, 5.2)	1.2 (0.9, 2.6)
Darunavir	5	60.1 (50.2, 66.5)	6.8 (6.2, 7.1)	3.5 (3.2, 3.9)
Lopinavir	2	41.6, 62.0	11.9, 4.9	2.4, 3.5
Ritonavir	12	4.9 (3.0, 9.4)	0.6 (0.4, 1.6)	0.2 (0.1, 0.4)
Efavirenz	3	24.5 (24.4, 68.2)	1.3 (1.1, 3.4)	1.3 (1.3, 3.4)

a *The range is reported for lopinavir and the median (range) is reported for efavirenz*.

Metabolic phenotyping was performed in 19 of 31 participants (12 HIV(–), 6 PI/r, and 1 EFV). The log ratio of DXM to DXO in the HIV(–) group was significantly higher than the PI/r group (−2.3 vs. −4.3, *p* = 0.01). Four of twelve participants in the HIV(–) group were CYP2D6 poor metabolizers, compared to zero of six PI/r participants. CYP2D6 phenotype was tested as a covariate on the sertraline population pharmacokinetic model, but did not significantly affect CL/F or V/F.

## Discussion

This is the first report of sertraline pharmacokinetics among youth living with HIV. Sertraline and N-desmethylsertraline exposure parameters trended lower or were significantly lower in the PI/r compared to the HIV(–) groups. Sertraline exposure, corrected for weight and dose, was much lower in EFV participants, but the sample size was very small. Few patients with psychiatric conditions were taking efavirenz, likely due to clinician concerns about central nervous system side effects associated with efavirenz use in patients with psychiatric disorders. The normalized sertraline AUC_0−24_ in the HIV(–) group (1,176 ng·hr/mL) was similar to that seen after multiple dosing in prior adult studies, normalized to a 100 mg dose (1038–1532 ng·hr/mL) (20). The normalized sertraline trough concentrations, C_0_ and C_24_, in the HIV(-) and PI/r groups of approximately 18–33 ng/mL were also similar to those in a therapeutic drug monitoring program, that reported a median and IQR sertraline trough with 100 mg daily doses of 20 ng/mL and 12–30 ng/mL (21). The observed troughs in the EFV group of 4.2 and 6 ng/mL were well below these typical trough values. The median weight-adjusted daily dose (mg/kg) in the HIV(–) group, while not statistically different, was higher than both groups of participants living with HIV. This suggests that clinicians are dosing sertraline more conservatively, possibly due to concerns about interacting antiretrovirals, in youth living with HIV. However, weight- and dose-normalized sertraline AUC_0−24_ suggests that youth taking common PI/r should be receiving equivalent, or potentially higher, weight-adjusted doses as their HIV-uninfected peers.

While we expected normalized sertraline exposures to be greater in HIV(–) vs. EFV participants, due to strong cytochrome P450 induction, we did not expect to also observe lower sertraline in PI/r participants. Sertraline is eliminated by multiple pathways including CYP2B6, CYP2D6, CYP2C9, CYP2C19, and CYP3A4. Ritonavir is a strong inhibitor of CYP2D6 and CYP3A4, but is also a strong inducer of CYP2C19 and UGT, a phase II metabolic pathway of sertraline (7). Involvement of numerous enzymes in sertraline metabolism make it unlikely for one agent to cause a clinically significant drug interaction (7, 20). Antiretroviral exposures in this study were similar to expected values, suggesting that participants were adherent to antiretrovirals. Our results, lower normalized (weight/dose) sertraline and N-desmethylsertraline AUC_0−24_ and the AUC ratio (N-desmethylsertraline/sertraline) in PI/r vs. HIV(–) participants, suggest that inhibition of various CYP enzymes by ritonavir does not increase sertraline exposure. Furthermore, decreased sertraline exposures in PI/r relative to HIV(–) were not a consequence of induction of enzymatic pathways, since ritonavir AUC_0−24_ did not correlate with the N-desmethylsertraline/sertraline AUC ratio. If induction was present, the AUC ratio would increase with increasing ritonavir. The most likely explanations for reduced sertraline exposures in the PI/r cohort are (1) decreased drug absorption in participants living with HIV and (2) lower sertraline doses prescribed (which can cause changes in sertraline pharmacokinetics that are not proportional to the difference in dose amount).

Another potential contributing factor to sertraline exposure differences could be underlying genetic polymorphisms in CYP enzymes. The Clinical Pharmacogenetics Implementation Consortium recommends a 50% decrease in sertraline dose in CYP 2C19 poor metabolizers due to increased adverse effects, but no change in dose for ultrarapid metabolizers. This recommendation is optional due to limited available evidence (22). CYP 2C19 genotypes of study participants were not determined.

A population pharmacokinetic study of geriatric patients (median age = 76 years) with Alzheimer's Disease also determined a 1-compartment model with first-order absorption and elimination best described sertraline, and estimated a clearance of 83.1 L/h (23). Our study estimated clearance at 115 L/h (for a typical 70 kg patient); 38% higher. In our model, of the covariates tested on CL/F and V/F (age, gender, race, cohort, CYP2D6 phenotype, aspartate aminotransferase, alanine aminotransferase, serum creatinine, and AUC ratio (N-desmethylsertraline/sertraline)), only age (independent of size) significantly affected clearance. This finding contrasts with previous pediatric studies and the aforementioned geriatric study, which found no associations between age and sertraline pharmacokinetics. Our finding should be interpreted with caution due to the limited range of ages in this study, 14–23 years. Gender did not affect sertraline pharmacokinetics in our study, similar to prior pediatric trials, but different from adults where men had an elimination half-life approximately 1.5 times greater than women (19).

EFV participants had sertraline exposures ~50% lower than HIV(–) and PI/r groups. In previous pediatric studies (15, 17), AUC_0−24_ (normalized to 100 mg dose and 70 kg weight) ranged from 883 to 2,535 ng·h/mL, much higher than 473 ng·h/mL in the EFV group. The metabolite-to-parent AUC ratio was approximately 50% higher in EFV than other cohorts, and efavirenz AUC was correlated with metabolite-to-parent AUC ratio (*r*^2^ = 0.97). These findings suggest that participants taking efavirenz have a much higher oral intrinsic clearance, possibly through induction of multiple CYP enyzmes that contribute to sertraline metabolism, including 2B6, 2C19, and 3A4. For 3A4 in particular, the reduction in EFV exposure could be due to induction at the intestinal level along with hepatic induction, resulting in both increased systemic clearance and decreased absorption due to first-pass loss upon oral administration. A larger sample size is needed to confirm this observation.

Participants in the HIV(–) group had sertraline weight-adjusted pharmacokinetic characteristics similar to adults. Ensuring dose modifications according to weight are therefore necessary for youth without HIV to achieve similar therapeutic exposures. Sertraline exposure in the PI/r cohort was ~30–40% lower compared to HIV(–). Concomitant ritonavir did not increase sertraline exposure as it does with many other medications, and youth taking a ritonavir-boosted protease inhibitor should be receiving at least the same sertraline dose (mg/kg), or potentially even modestly higher doses, as uninfected youth and adults. Participants taking efavirenz had much lower sertraline exposures than the two other cohorts and adults. Even though psychiatric medications are often titrated to effect, these participants did not receive higher absolute or weight-adjusted doses than the other groups, and likely had sub-therapeutic exposures. Caution should be exercised when interpreting findings from the EFV cohort due to the small sample size (*n* = 3), but higher doses or therapeutic drug monitoring of sertraline in this population may be warranted.

## Data Availability Statement

The data cannot be made publicly available due the ethical restrictions in the study's informed consent documents and in the International Maternal Pediatric Adolescent AIDS Clinical Trials (IMPAACT) Network's approved human subjects protection plan; public availability may compromise participant confidentiality. However, data are available to all interested researchers upon request to the IMPAACT Statistical and Data Management Center's data access committee (email address: sdac.data@fstrf.org) with the agreement of the IMPAACT Network.

## Ethics Statement

All participants gave written informed consent, permission or assent in accordance with the Declaration of Helsinki and local guidelines. The protocol was approved by all relevant ethics or human subjects protection committees at all participating sites.

## Author Contributions

All co-authors reviewed, revised for content, and approved this article. MP, YH, SK, ES, GS, PB, EC, and BB: substantial contributions to the conception and design; NH, MP, BG, and BJ: acquisition of data; NH, MP, YH, SK, ES, GS, PB, EC, and BB: analysis or interpretation of data.

### Conflict of Interest Statement

The authors declare that the research was conducted in the absence of any commercial or financial relationships that could be construed as a potential conflict of interest.
